# Association between nickel exposure and diabetes risk: an updated meta-analysis of observational studies

**DOI:** 10.3389/fpubh.2024.1463880

**Published:** 2024-11-05

**Authors:** Huaye Lu, Xiaoyang Shi, Lei Han, Xin Liu, Qingtao Jiang

**Affiliations:** ^1^Institute of Occupational Disease Prevention, Jiangsu Provincial Center for Disease Control and Prevention (Jiangsu Provincial Academy of Preventive Medicine), Nanjing, China; ^2^Department of Clinical Medicine, Jiangsu Health Vocational College, Nanjing, China

**Keywords:** nickel, heavy metal, diabetes, hyperglycemia, meta-analysis

## Abstract

**Objective:**

The results of epidemiological studies on the association between nickel exposure and diabetes remain controversial. Therefore, an update meta-analysis was conducted to examine the association between urinary nickel levels and diabetes risk, and to focus on whether there is an association between blood nickel levels and diabetes risk.

**Methods:**

Relevant studies were comprehensively searched from PubMed, Web of Science, and Wanfang databases from their inception to July 2024. The random-effects model was utilized to determine pooled Standard Mean Difference (SMD) and 95% confidence intervals (CIs), with stratified and sensitivity analyses also performed. Heterogeneity between studies was assessed using *I*^2^ statistic, while publication bias was evaluated using Egger's and Begg's tests. The quality of the included studies was assessed using the Newcastle-Ottawa Scale.

**Results:**

A total of 19 studies involving 46,071 participants were included in this meta-analysis. The random-effects model indicated that the pooled SMD for nickel exposure levels in diabetic patients and non-diabetic controls were 0.16 (95% CI 0.07–0.2) for urine and 0.03 (95% CI −0.20 to 0.27) for blood, respectively.

**Conclusion:**

It was discovered that diabetes risk was positively correlated with urinary nickel levels, whereas there was no significant correlation with blood nickel levels. Furthermore, it appeared that the association between nickel exposure and diabetes risk differ in individuals with diabetes compared to those with pre-diabetes, and that the direction of the correlation may even be reversed. In conclusion, more high-quality prospective studies are needed in order to validate these findings in future research endeavors.

**Systematic Review Registration:**

https://www.crd.york.ac.uk/PROSPERO, registration number: CRD42024534139.

## 1 Introduction

Diabetes mellitus (DM), commonly known as diabetes, is a chronic metabolic disease that leads to persistently high blood glucose levels due to inadequate insulin secretion or insulin action (1). The prevalence of diabetes presents an increasing global burden for individuals, families, and countries. According to the IDF Diabetes Atlas (2021)^1^, 10.5% of the adult population (20–79 years) has diabetes. Furthermore, new projections published in The Lancet suggest that more than 1.31 billion people worldwide may be affected by diabetes by 2050, with type 2 diabetes accounting for 90% of all cases (2). Major risk factors for type 2 diabetes include high Body Mass Index (BMI), genetics, dietary risks, environmental and occupational risks, and unhealthy lifestyle choices. Several studies highlight the inequity of diabetes, forecasting that by 2045, up to three-quarters of diabetic persons will live in low-income and middle-income nations (2–4). These populations are particularly susceptible to environmental and occupational risks which can accumulate in human organ tissues and induce chronic toxicity. Therefore it is crucial to investigate their impact on diabetes.

Metal contamination poses a significant threat in terms of environmental and occupational risks, and cannot be overlooked. Certain metals, such as zinc, cadmium, nickel, and mercury have been associated with the development of type 2 diabetes (5, 6). Nickel, a naturally occurring element in food, represents the primary source of exposure for the average individual. However, in recent years, nickel has gained widespread industrial application due to its corrosion resistance, physical strength, and specific magnetic electronic capabilities, which greatly increases the potential for human exposure through environmental pollution and occupational hazards. Previous studies have indicated that nickel, as an external endocrine disruptor, may play a crucial role in metabolic disorders, including altering glucose metabolism and insulin homeostasis (7).

Nickel has been shown in animal studies to induce hyperglycemia, glucagon, and insulin resistance (8–11). However, the effects of nickel exposure on human diabetes observed in population researches were inconsistent (12). Several prior investigations in adults around the world have found that higher levels of urinary nickel are associated with an increased risk of diabetes (13–15). Similarly, a large sample of monitoring data from China indicated that each one-unit increase in log-transformed urine nickel concentrations was linked to a 0.36 (0.17, 0.55 mmol/L) increase in fasting blood glucose (FBG) (16). Two additional studies of Chinese adults with diabetes confirmed this finding (17, 18). Similarly, the Study of Women's Health Across the Nation in US found no statistical association between urinary nickel and diabetes (19). Furthermore, the effect of other biomarkers (such as blood nickel) on diabetes risk is also controversial. One investigation examining associations of multiple plasma metals with incident type 2 diabetes in Chinese adults found no significant relationship between blood nickel and diabetes (20). Whereas another case-control study found that blood nickel may contribute to the development of diabetes (OR = 2.24) (14).

A previously published meta-analysis revealed a significant linear dose-response association between nickel exposure in urine and the risk of diabetes. However, no meaningful results were found between blood nickel levels and diabetes risk. The study suggests that urinary nickel levels are more reliable biomarkers of exposure (21). However, this investigation only pooled three articles on blood nickel and diabetes risk, which may have influenced the conclusions. Therefore, given the uncertainty in the existing literature regarding the association between nickel exposure and diabetes risk, we conducted this updated meta-analysis to examine the association between urinary nickel levels and diabetes risk, and to focus on whether there is an association between blood nickel levels and diabetes risk.

## 2 Methods

### 2.1 Literature search

Two independent investigators (Huaye Lu and Xiaoyang Shi) conducted a comprehensive literature search of the PubMed, Web of Science, and Wanfang databases from their establishment to July 2024. The search terms used were “nickel” or “heavy metals” as well as “diabetes” or “impaired glucose tolerance” or “impaired fasting glucose.” Supplementary Table S1 summarizes the detailed search methodologies, which is listed in the Supplementary material. Furthermore, the reference lists of relevant material were reviewed to guarantee a complete search of all relevant literature on the issue. The linguistic limitations for inclusion were English and Chinese. All retrieved articles were imported into EndNote (version X9.1), where duplicates were deleted. Two investigators (Huaye Lu and Xiaoyang Shi) independently evaluated all titles and abstracts, as well as conducting a full-text evaluation of the included reports. Disagreements were resolved by discussions with a third investigator (Xin Liu).

### 2.2. Study inclusion criteria

The studies included in our study met the following inclusion criteria: (1) observational studies, including cohort, case–control, or cross-sectional studies; (2) the exposure under investigation was the concentration of nickel in the blood or urine; (3) the outcome of interest was type 2 diabetes or impaired fasting glucose (IFG) or impaired glucose tolerance (IGT) or raised glycated hemoglobin (HbA1c); (4) the sample sizes for the case and control groups, as well as the mean and standard deviation of nickel concentrations in the associated biological samples, were either published or can be calculated using actual raw data; (5) when data from the same population was published multiple times, the most recent and comprehensive data was chosen.

### 2.3. Risk of bias assessment

The risk of bias in the included studies were assessed using the Newcastle-Ottawa Scale. The Cochrane Collaboration recommends using this tool to assess the likelihood of bias in observational studies (22). The total score is 9 points, with 0–4 indicating poor quality literature, 5–7 indicating medium-level literature, and 8–9 indicating good quality literature. To avoid excessive bias, low-quality literature was omitted from the meta-analysis results.

### 2.4. Data extraction

We collected the following information from the eligible studies: (1) first author's name; (2) year of publication; (3) language; (4) study region; (5) study design; (6) type of exposure assessment; (7) number of cases and control; (8) gender distribution; (9) age range or mean age; (10) nickel exposure levels; (11) outcomes of interest; (12) methods for outcome assessment. If additional information is required, we will contact the original article's author.

### 2.5. Statistical analysis

In this meta-analysis, we measured the relationship between nickel exposure and diabetes risk by comparing mean and standard deviation across different groups. Given that many data points in the real study did not follow the normal distribution, QR was utilized to approximate SD (SD = QR/1.35) when the median and quartile spacing were employed to represent nickel concentration in the study. The Cochran's *Q*-test and the *I*^2^ statistic were used to assess study heterogeneity (*I*^2^ values of 25%, 50%, and 75% indicate low, moderate, and high heterogeneity, respectively). Given the substantial heterogeneity of observational research, all analyses were conducted using a more cautious random effects model. Sensitivity analysis was performed to determine whether eliminating one study at a time had a substantial impact on the outcome. The funnel plot was used to analyze publication bias qualitatively, while Egger's and Begg's tests were used to assess it statistically. R (Version 4.3.1) software was used to conduct statistical analyses on all data. All tests were two-sided, and *P* < 0.05 indicated statistical significance.

## 3 Results

### 3.1 Study screening

**Figure 1:**
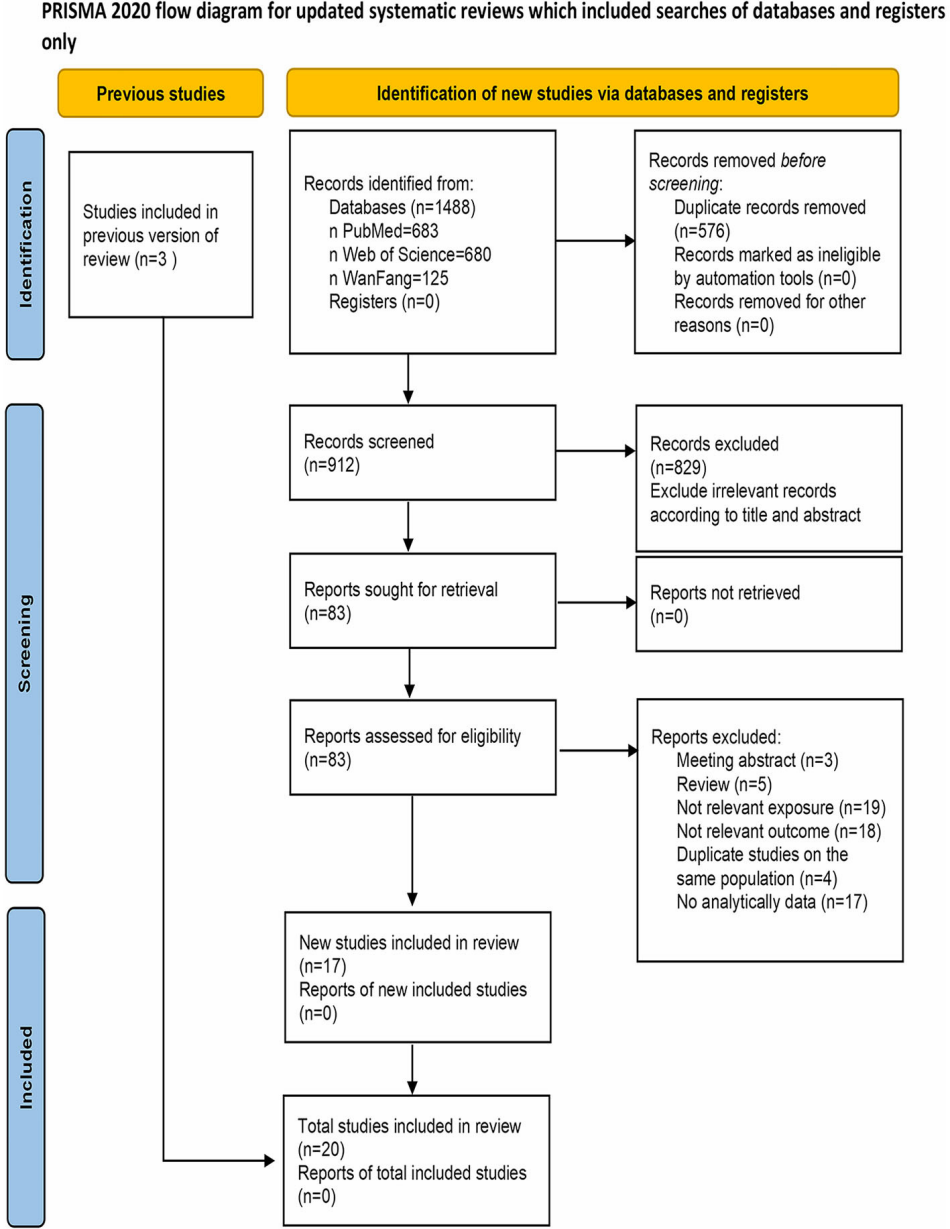
Flow diagram of the literature search and selection process.

The PRISMA 2020 flow diagram depicting the literature search is presented in Figure 1. Initially, a total of 1,488 records were retrieved. After removing duplicates, 912 records remained. Subsequently, 83 studies were identified as relevant after reviewing titles and abstracts. Upon full-text assessment, 66 studies were further excluded for reasons such as lack of inclusion of nickel in the exposure assessment (*n* = 19), non-diabetes related outcomes (*n* = 18), duplicate studies on the same population (*n* = 4), unavailability or unsuitability of data (*n* = 17). Additionally, meeting abstracts (*n* = 3) and reviews (*n* = 5) were also excluded. The remaining 17 studies were deemed qualified for inclusion. Three additional studies were added through a reverse citation search of the included studies. Ultimately, a total of 20 studies were included in this meta-analysis.

### 3.2 Characteristics of the included studies and risk of bias evaluation

Next, we evaluated the risk of bias in 20 publications and excluded one due to low quality. The study includes 19 eligible literatures, consisting of 9 (47.4%) high-quality and 10 (52.6%) medium-quality literatures (11, 14, 17, 18, 21–34). The results of literature quality assessment are provided in Supplementary Table S2, and specific information on all literatures can be found in Supplementary Table S3.

Overall, the majority of the 19 publications included were from Asia (*n* = 15), with a few from the Americas (*n* = 2) and Europe (*n* = 2), which were published between 2000 and 2024. Nine studies used urinary nickel concentrations to determine exposure, with one study separated into diabetes and hyperglycemia groups, yielding a total of 10 sets of data. Eleven studies used blood nickel concentrations to determine exposure, resulting in 15 sets of data.

### 3.3 Urinary nickel and diabetes risk

**Figure 2:**
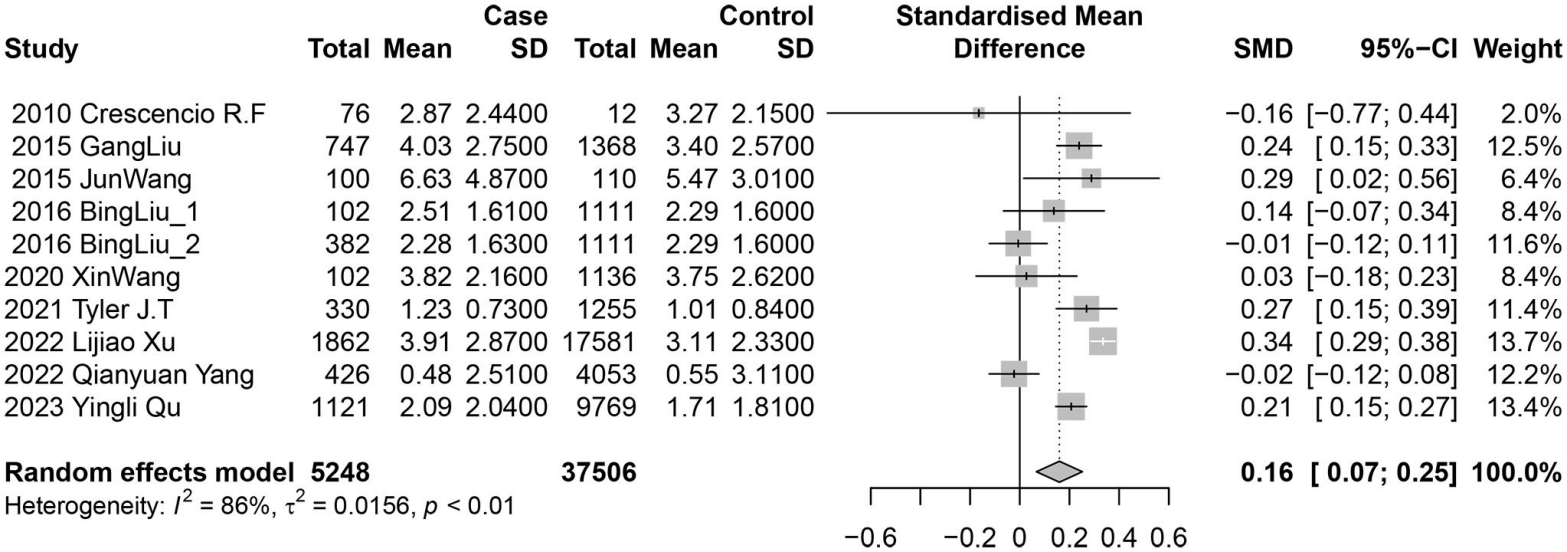
Forest plot of the associations between urinary nickel levels and diabetes risk.

The studies were categorized into urinary nickel and blood nickel groups. Nine studies (10 data sets) examined the association between urinary nickel levels and diabetes risk. Among them, five reported a positive and statistically significant relationship, while the other five found no significant associations. The random-effects model indicated a positive link between diabetes risk and nickel exposure levels, with a pooled SMD of 0.16 (95% CI 0.07–0.25; *I*^2^ = 86%; *P* heterogeneity <0.01) for urine (Figure 2).

Given the substantial heterogeneity in studies on urinary nickel and diabetes, a meta-regression analysis was conducted to examine various study characteristics. The findings indicated that study region, study design and language did not have a significant impact on the heterogeneity between studies (Table 1).

**Table 1:** Meta-regression analysis of urinary nickel and diabetes risk.

**Covariable**		**β (95%CI)**	***Z*-value**	***P***
Study region	China	—	—	—
	Mexico	−0.36 (−1.01, 0.29)	−1.08	0.28
	USA	0.07 (−0.19, 0.34)	0.55	0.58
Study design	Cohort	—	—	—
	CS	0.20 (−0.01, 0.41)	1.83	0.07
Language	Chinese	—	—	—
	English	−0.09 (−0.46, 0.27)	−0.51	0.61

China, cohort and Chinese were used as reference group.

CS, cross-sectional study.

### 3.4 Blood nickel and diabetes risk

Eleven studies (15 data sets) investigated the association between blood nickel levels and diabetes risk. Three reported positive results, four reported negative results, and the other eight showed no significant connections. In the random-effects model, there were no significant relationships between diabetes risk and nickel exposure levels, with a pooled SMD of 0.03 (95% CI −0.20 to 0.27; *I*^2^ = 83%; *P* heterogeneity <0.01) for blood.

**Figure 3:**
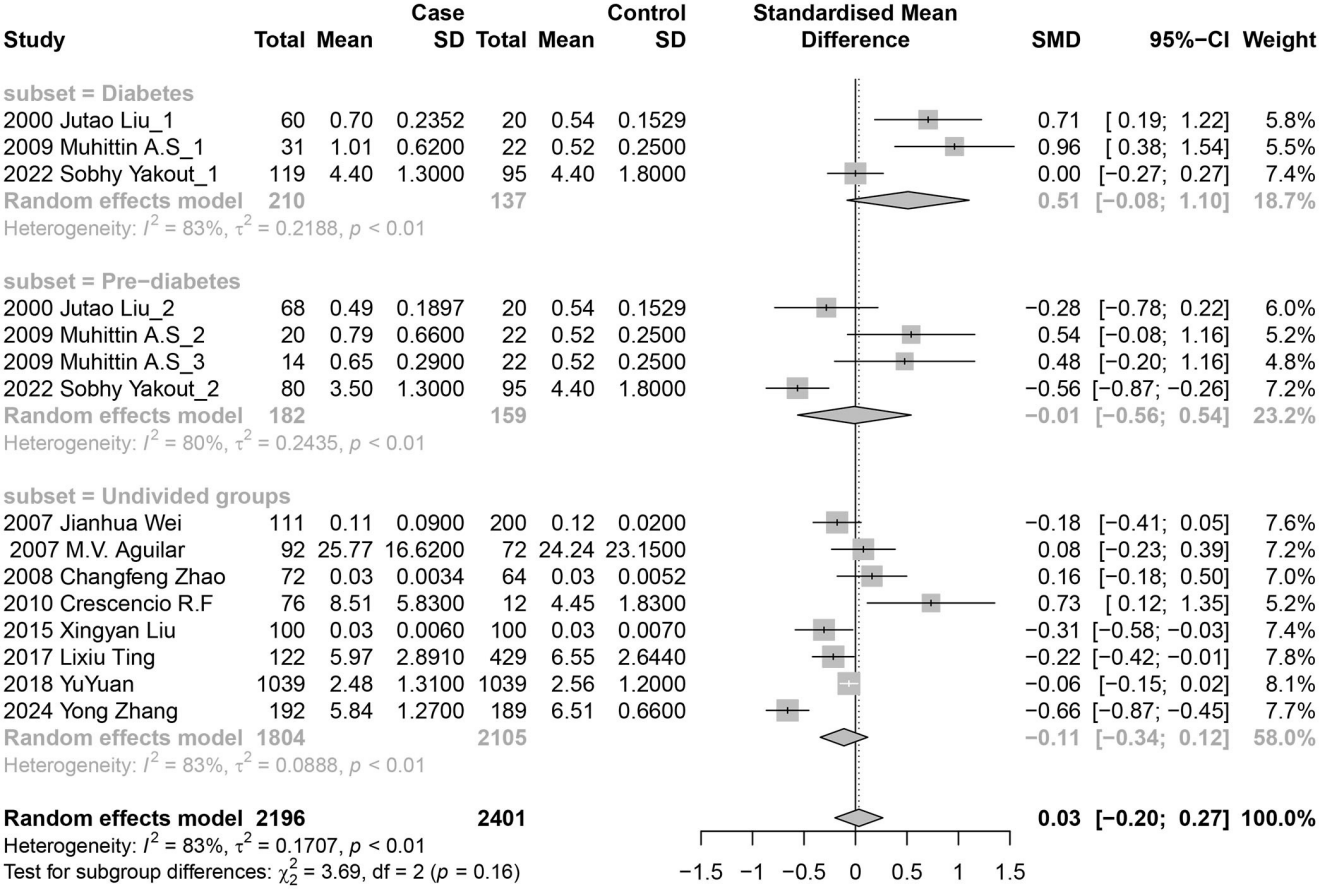
Forest plot of the associations between blood nickel levels and diabetes risk. Subgroup analysis was performed according to the progression of diabetes.

Subsequently, we conducted a subgroup analysis (Figure 3): individuals with diabetes were classified as Diabetes groups, those with IGT or IFG were classified as Pre-diabetes groups, which had not been previously divided in the literature was classified as Undivided groups. The findings of subgroup analysis revealed no statistical difference among groups, including the Diabetes groups with a pooled SMD of 0.51 (95% CI −0.08 to 1.10), Pre-diabetes groups with a pooled SMD of −0.01 (95% CI −0.56 to 0.54), and Undivided groups with a pooled SMD of −0.11 (95% CI −0.34 to 0.12).

### 3.5. Sensitivity analyses and publication bias

**Figure 4:**
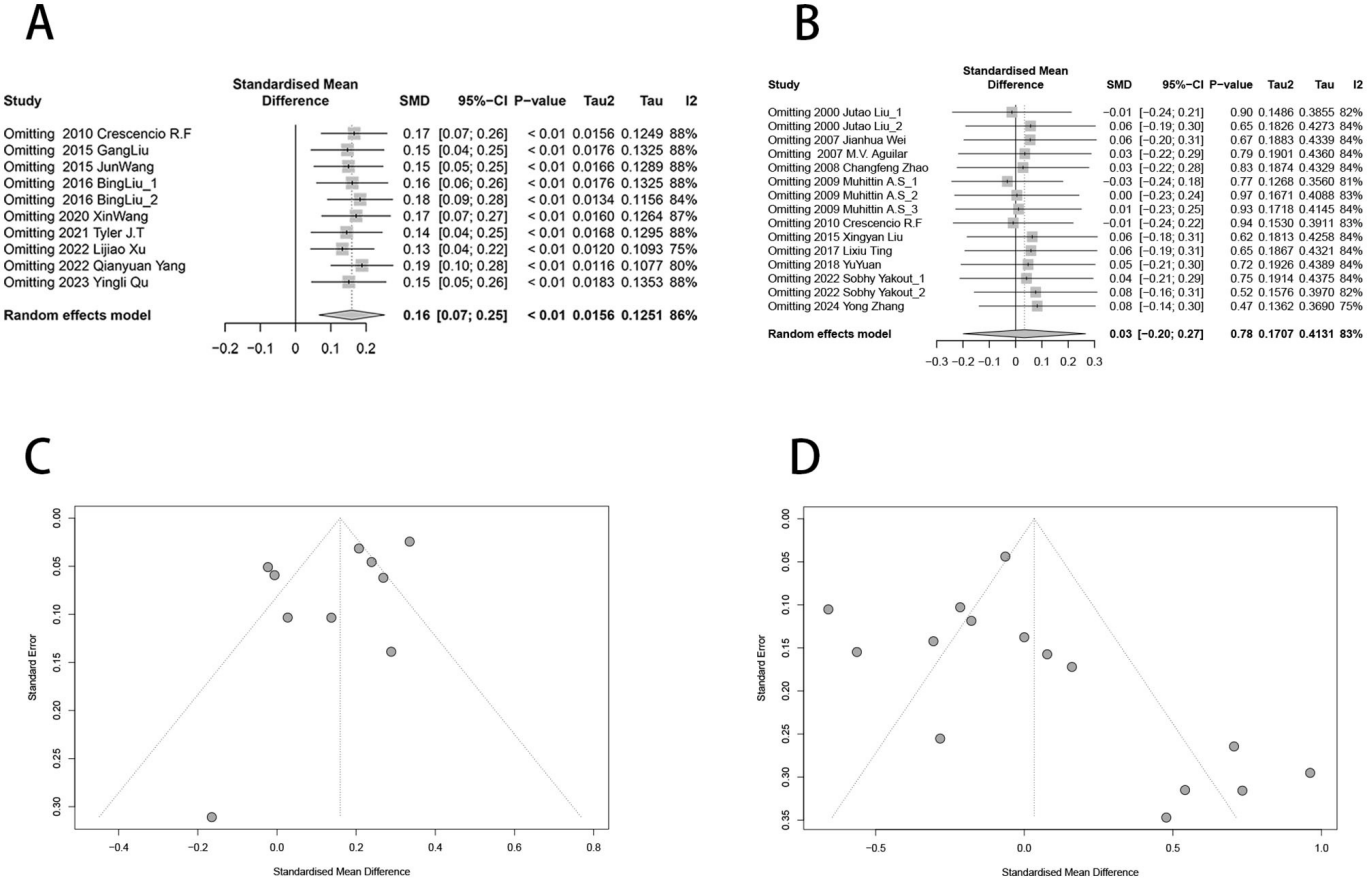
Funnel plot and sensitivity analysis of urinary nickel, blood nickel and diabetes risk. **(A)** Sensitivity analysis of urinary nickel levels and diabetes risk; **(B)** Sensitivity analysis of blood nickel levels and diabetes risk; **(C)** Funnel plot of urinary nickel and diabetes risk; **(D)** Funnel plot of blood nickel and diabetes risk.

Sensitivity analysis was conducted to assess the impact of individual studies on the overall effect for urinary nickel, blood nickel, and diabetes risk by excluding specific studies. The results showed that after removing any individual studies, there were no statistically significant differences in the effect estimates (Figure 4). Additionally, the funnel plots displayed approximate symmetry (Figure 4). Egger's test (*t* = −1.77, *P* = 0.115) and Begg's test (*z* = −0.45, *P* = 0.655) indicated no significant publication bias for urine (Supplementary Figures S1a, S2a), while Egger's test (*t* = 1.23, *P* = 0.240) and Begg's test (*z* = 2.33, *P* = 0.02) were inconsistent for blood (Supplementary Figures S1b, S2b).

## 4 Discussion

This analysis comprised three cohort studies, two case-control studies, and 14 cross-sectional studies involving a total of 46,071 participants. Overall, the meta-analysis revealed a weak positive correlation between urinary nickel levels and diabetes risk (SMD is 0.16), while no such association was found with blood nickel levels. Physiologically it is plausible that having elevated levels of nickel in urine may elevate the risk of developing diabetes. As previous research has shown that nickel can induce hyperglycemia through increased hepatic glycogenolysis, heightened pancreatic glucagon release, reduced peripheral utilization of glucose or gluconeogenesis (37). Furthermore, nickel may also elevate inducible nitric oxide synthase and cyclic guanosine monophosphate to induce hyperglycemia (5). A previous meta-analysis demonstrated that for the highest vs. lowest urinary nickel exposure category, there was a positive association between nickel and diabetes with pooled ORs of 1.42 (95% CI: 1.14–1.78) (21), which aligns with our findings. In addition, Qu et al. reported that the relationship between urinary nickel levels and diabetes risk was significant, and after excluding individuals with extremely high nickel exposure, the association between urinary nickel exposure and diabetes was significant, monotonic, and increasing (16). The dose-response analysis conducted by Xia et al. also found that each 1 μg/L increase in urinary nickel concentration was related to a 7% increase in the risk of developing diabetes (21). These conclusions appear to support urinary nickel as a reliable biomarker of exposure to diabetes.

On the other hand, our findings revealed no significant link between blood nickel levels and diabetes risk. The available literature is indeed divided on this issue. A population-based study in Norway found that blood nickel levels were greater in diabetic patients (*N* = 128) than in non-diabetic controls (*N* = 755) (14). However, another cohort study from China found that diabetic patients (*N* = 1,039) had lower blood nickel concentrations than non-diabetic controls (*N* = 1,039) (20).

However, we have observed an interesting phenomenon in the three studies we included. These studies grouped the subjects according to their diabetes status and all found that the blood nickel concentration in the prediabetic groups was lower than that in the diabetic groups (23, 27, 35). Additionally, two of these studies even found that the blood nickel concentration in the prediabetic groups was lower than that in the healthy groups. This phenomenon was also observed in a study belonging to the urinary nickel groups, which stratified the subjects based on their diabetes status (32). The urinary nickel concentrations were 2.51 (1.48–3.66) μg/L and 2.29 (1.41–3.57) μg/L in the diabetic and healthy groups respectively, but 2.28 (1.33–3.53) μg/L in the hyperglycemic group. The variation of blood nickel concentrations over different stages of diabetes may explain why previous studies without patient stratification have yielded inconsistent results or even opposite effects. Existing mechanistic research has reported that administration of nickel chloride could prevent alloxan or streptozotocin-induced hyperglycemia by increasing Cu-Zn superoxide dismutase activity, suggesting a protective effect against hyperglycemia (5). Furthermore, a population-based study have indicated a protective effect of plasma nickel concentrations within a specific range against type 2 diabetes mellitus risk, nickel may have a dual effect on the risk of T2DM, with a protective range of <6.1μg/L (36).

In light of this thought, we conducted a stratified analysis of blood nickel groups based on the stage of diabetes. Unfortunately, the results of the stratification were still not statistically significant. However, the effect size changed from 0.03 (−0.20, 0.27) to 0.51 (−0.08, 1.10), with the lower 95%CI approaching 1 in the Diabetes groups. Due to the limited number of eligible studies, particularly those that stratify patients with diabetes, it is uncertain whether the lack of positive results is due to insufficient power of test. Nevertheless, based on existing research, we believe that this is a scientific question worthy of further investigation.

One major advantage of our study is the high number of participants, which significantly reduces sampling error and increases the likelihood of drawing reasonable conclusions. Another advantage is sensitivity analysis revealed that the results were steady, and there was no significant publication bias. However, there are several limitations to this meta-analysis. Firstly, the majority of included studies were cross-sectional, thus temporal relationships could not be established. More prospective studies are needed to verify this association. Secondly, there is significant heterogeneity between studies, in terms of offering suggestions for future research, the direction of our research is not to fully explain its sources. As previously mentioned, the evidence for a relationship between urinary nickel as a biomarker and diabetes is relatively strong, therefore our conclusions can serve as a supplement with limited discussion. The most significant finding of our study is that blood nickel concentration appear to be different on patients with pre-diabetes and diabetes, suggesting that the effect of nickel on diabetes may not be linear or even dual in nature. This provides a meaningful clue for future research direction. In the future, more mechanism-based and population-based studies are required to further explore this issue in order to address research gaps in this field and elucidate their relationship more comprehensively.

In conclusion, our study has indicated a positive association between urinary nickel levels and diabetes risk. However, when assessed using blood levels, the link between blood nickel levels and diabetes risk was not found to be statistically significant. Furthermore, we observed that the association between nickel exposure and diabetes risk appears to differ in individuals with diabetes compared to those with pre-diabetes, and that the direction of the correlation may even be reversed. It is important to note that since cross-sectional studies comprise most of the research included in our meta-analysis, we cannot establish a definitive cause-and-effect relationship based solely on these findings. Additionally, any role of nickel in diabetes is likely not a single effect but may interact with various other factors. Therefore, more high-quality studies are needed to confirm whether nickel exposure impacts diabetes and further understand how this potential effect operates.

## Data Availability

The original contributions presented in the study are included in the article/Supplementary material, further inquiries can be directed to the corresponding authors.

## Footnotes

^1^ https://diabetesatlas.org/
