# Supplementary material for: Association between nickel exposure and diabetes risk: an updated meta-analysis of observational studies
Source: Front Public Health. 2024 Nov 5;12:1463880. doi: 10.3389/fpubh.2024.1463880 (PMC11574963; doi:10.3389/fpubh.2024.1463880)

Supplementary Material

# Supplementary Tables

| **Table S1.** Detailed search strategies | | |
| --- | --- | --- |
| Databases | Search strategies | Results |
| PubMed | ("heavy metal*"[All Fields] OR ("nickel"[MeSH Terms] OR "nickel"[All Fields] OR "nickelous"[All Fields] OR "nickels"[All Fields])) AND ("diabete*"[All Fields] OR "impaired fasting glucose"[All Fields] OR "impaired glucose tolerance"[All Fields] OR "glucose intolerance"[All Fields]) | 683 |
| Web of Science | TS=(("heavy metal*" OR nickel) AND (diabete* OR "impaired fasting glucose" OR "impaired glucose tolerance" OR "glucose intolerance")) | 680 |
| WanFang | (Title or key words: (nickel) or all: (heavy metals) or all: (nickel element)) and (title or key words: (diabetes) or all: (impaired fasting blood glucose) or all: (abnormal glucose tolerance) or all: (abnormal fasting blood glucose)) | 125 |
| ALL |  | 1488 |

**Table S2.** Summary of using the NOS scale to estimate the risk of overall study level bias

| Studies | 1-1 | 1-2 | 1-3 | 1-4 | 2-1 | 3-1 | 3-2 | 3-3 | Total NOS score |
| --- | --- | --- | --- | --- | --- | --- | --- | --- | --- |
| 2000 Jutao Liu | 0 | 1 | 0 | 1 | 0 | 1 | 1 | 1 | 5 |
| 2007 Jianhua Wei | 1 | 1 | 1 | 1 | 0 | 1 | 1 | 1 | 7 |
| 2007 M.V. Aguilar | 1 | 0 | 1 | 1 | 2 | 1 | 1 | 1 | 8 |
| 2008 Changfeng Zhao | 1 | 1 | 1 | 1 | 0 | 1 | 1 | 1 | 7 |
| 2009 Muhittin A.S | 1 | 0 | 0 | 1 | 0 | 1 | 1 | 1 | 5 |
| 2010 Crescencio R.F | 1 | 0 | 0 | 1 | 0 | 1 | 1 | 1 | 5 |
| 2012 HuiChen | 0 | 0 | 0 | 0 | 0 | 1 | 1 | 1 | 3 |
| 2015 GangLiu | 1 | 1 | 1 | 1 | 2 | 1 | 1 | 0 | 8 |
| 2015 Xingyan Liu | 1 | 1 | 0 | 1 | 0 | 1 | 1 | 1 | 6 |
| 2015 JunWang | 1 | 0 | 1 | 1 | 0 | 1 | 1 | 1 | 6 |
| 2016 BingLiu | 1 | 1 | 0 | 1 | 2 | 1 | 1 | 0 | 7 |
| 2017 Lixiu Ting | 1 | 1 | 1 | 1 | 1 | 1 | 1 | 1 | 8 |
| 2018 YuYuan | 1 | 1 | 1 | 1 | 2 | 1 | 1 | 1 | 9 |
| 2020 XinWang | 1 | 1 | 1 | 1 | 2 | 1 | 1 | 1 | 9 |
| 2021 Tyler J.T | 1 | 1 | 1 | 1 | 2 | 1 | 1 | 1 | 9 |
| 2022 Lijiao Xu | 1 | 1 | 1 | 1 | 2 | 1 | 1 | 0 | 8 |
| 2022 Sobhy Yakout | 1 | 0 | 0 | 1 | 0 | 1 | 1 | 1 | 5 |
| 2022 Qianyuan Yang | 1 | 1 | 1 | 1 | 2 | 1 | 1 | 1 | 9 |
| 2023 Yingli Qu | 1 | 1 | 1 | 1 | 2 | 1 | 1 | 1 | 9 |
| 2024 Yong Zhang | 1 | 1 | 1 | 1 | 2 | 1 | 1 | 1 | 9 |

***Annotation:*** the total score is 9 points, with 0-4 indicating poor quality literature, 5-7 indicating medium-level literature, and 8-9 indicating good quality literature.

**Table S3.** Basic characteristics of the studies included in the meta-analysis

| Studies | Study  region | Study  design | Statistical  method | Population  characteristics | M(Q)/X±S(cases) | M(Q)/X±S (control) | Outcome  assessment |
| --- | --- | --- | --- | --- | --- | --- | --- |
| 2000 Jutao Liu | China | CS | ANOVA | 148 individuals (68 T2DM, 60 hyperglycemia, 20 non-DM), 72 males and 76 females | T2DM: 0.6978±0.2352mg/L hyperglycemia: 0.4904±0.1897mg/L | 0.5424±0.1529mg/L | self-description |
| 2007 Jianhua Wei | China | CS | t-test | 311 individuals aged 27–84 years (111 T2DM, 200 non-DM) | 0.11±0.09mg/L | 0.12±0.02mg/L | 1985 WHO diagnostic criteria for diabetes mellitus |
| 2007 M.V. Aguilar | Spain | CS | ANOVA, LMR | 164 individuals (92 T2DM with mean age 60.3, 72 non-DM with mean age 62.4) | 25.77±16.62nmol/L | 24.24±23.15nmol/L | self-description |
| 2008 Changfeng Zhao | China | CS | t-test | 136 individuals (72 T2DM with mean age 60.3, 64 non-DM with mean age 63.1) | 0.0282±0.0034mg/L | 0.0275±0.0052mg/L | 1999 WHO diagnostic criteria for diabetes mellitus |
| 2009 Muhittin A.S | Turkey | CS | M-W U test | 87 individuals (31 T2DM, 20 IGT, 14 IFG, 22 non-DM) | T2DM: 1.01±0.62ug/L IGT：0.79±0.66ug/L IFG：0.65±0.29ug/L | 0.52±0.25ug/L | T2DM: FPG≥11.0mmol/L  IGT: FPG 7.0-11.0 mmol/L  IFG: FPG 5.6-6.9 mmol/L |
| 2010 Crescencio R.F | Mexico | CS | t-test | 88 individuals (76 T2DM, 12 non-DM) | 8.51±5.83ug/L | 4.45±1.83ug/L | DM treatment (physician-based) |
| 2015 GangLiu | China | CS | ANCOVA, LRM | 2115 individuals (747 T2DM, 1368 non-DM), 896 males and 1219 females | 4.03(2.60-6.31) ug/L | 3.40(2.19-5.66) ug/L | T2DM: FPG≥7.0mmol/L |
| 2015 Xingyan Liu | China | CS | t-test | 200 individuals (100 T2DM, 100 non-DM), 112 males and 88 females | 0.029±0.006mg/L | 0.031±0.007mg/L | T2DM: FPG≥7.0mmol/L |
| 2015 JunWang | China | CS | Wilcoxon r-s test | 210 individuals (100 T2DM, 110 non-DM), 118 males and 92 females | 6.63(4.26-10.83) ug/g | 5.47(3.91-7.79) ug/g | 1997 American Diabetes Association diagnostic criteria for diabetes |
| 2016 BingLiu | China | CS | t-test, LRM | 1493 individuals (102 T2DM, 382 hyperglycemia, 1111 non-DM) | T2DM: 2.51(1.48-3.66)ug/L  Hyperglycemia: 2.28(1.33-3.53) ug/L | 2.29(1.41-3.57)ug/L | T2DM: FPG≥7.0mmol/L  IFG: FPG 5.6-6.9 mmol/L |
| 2017 Lixiu Ting | China | CC | ANOVA | 551 individuals (122 T2DM, 429 non-DM), 216 males and 335 females | 5.968(3.903) ug/L | 6.551(3.570) ug/L | T2DM: FPG≥7.0mmol/L or HbA1c≥6.5% |
| 2018 YuYuan | China | cohort | M-W U test | 2078 individuals (1039 T2DM with mean age 62.8, 1039 non-DM with mean age 62.9) | 2.48 (1.86, 3.63)ug/L | 2.56 (1.95, 3.57) ug/L | T2DM: FPG≥6.1mmol/L |
| 2020 XinWang | China | cohort | COX | 1138 individuals (102 T2DM with mean age 50.0, 1136 non-DM with mean age 49.5) | 3.82 (2.58–5.50)ug/L | 3.75 (2.27–5.81)ug/L | T2DM: FPG≥126 mg/dL |
| 2021 Tyler J.T | USA | CS | ANOVA | 1585 individuals (330 T2DM, 1255 non-DM) | 1.23 (0.81-1.80)ug/L | 1.01 (0.57-1.71)ug/L | T2DM: FPG≥126 mg/dL or HbA1c≥6.5% |
| 2022 Lijiao Xu | China | CS | ANCOVA, LRM | 18443 individuals (1862 T2DM with mean age 58.8, 17581 non-DM with mean age 63.0) | 3.91 (2.47–6.34) ug/g | 3.11 (1.94–5.09) ug/g | T2DM: DM treatment (physician-based) or random glucose ≥11.1 mmol/L |
| 2022 Sobhy Yakout | Saudi | CS | ANOVA、  K-W test | 294 individuals (119 T2DM, 80 pre-diabetes, 95 non-DM) | T2DM: 4.4 (3.6-5.3)ug/L  pre-diabetes: 3.5 (2.2-4.0) ug/L | 4.4 (3.5-5.9)ug/L | T2DM: FPG > 7.0mmol/L  pre-diabetes: FPG 6.1-7.0 mmol/L |
| 2022 Qianyuan Yang | China | cohort | LRM | 4455 individuals (426 T2DM, 4053 non-DM), 1589 males and 2890 females | 0.48 (0.00–3.39)ug/L | 0.55 (0.00–4.20) ug/L | T2DM: FPG≥7.0mmol/L or HbA1c≥6.5% |
| 2023 Yingli Qu | China | CS | LRM | 10890 individuals (1121 T2DM, 9769 non-DM), 5462 males and 5446 females | 2.09(1.07-3.82)ug/L | 1.71(0.83-3.28)ug/L | T2DM: FPG≥7.0mmol/L |
| 2024 Yong Zhang | China | CC | M-W U test, LRM | individuals (192 T2DM with mean age 55.5, 189 non-DM with mean age 53.0) | 5.84 (4.88–6.59) ug/L | 6.51 (5.69–7.40)ug/L | T2DM: FPG≥7.0mmol/L or random glucose ≥11.1 mmol/L or HbA1c≥6.5% |

***Annotation:*** CS: cross-sectional study, CC: case–control study, LRM: logistic regression model, COX: cox proportional-hazards model, DM: Type 2 diabetic subjects, non-DM: non diabetic subjects, FPG: fasting plasma glucose, IFG: impaired fasting glucose, HbA1c: glycosylated hemoglobin

# Supplementary Figures

**Figure S1a, S1b.** Egger's publication bias plot of urinary nickel, blood nickel and diabetes risk


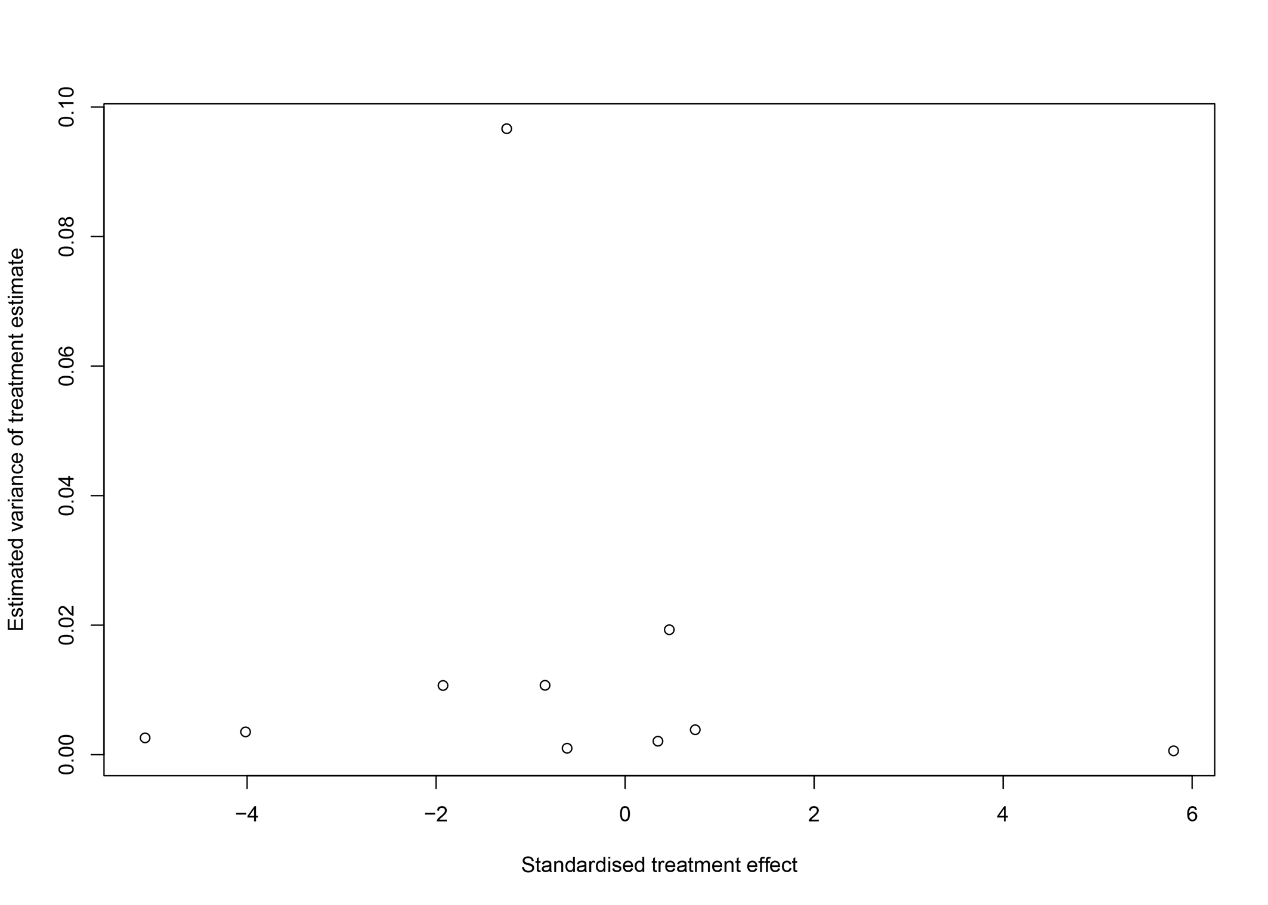


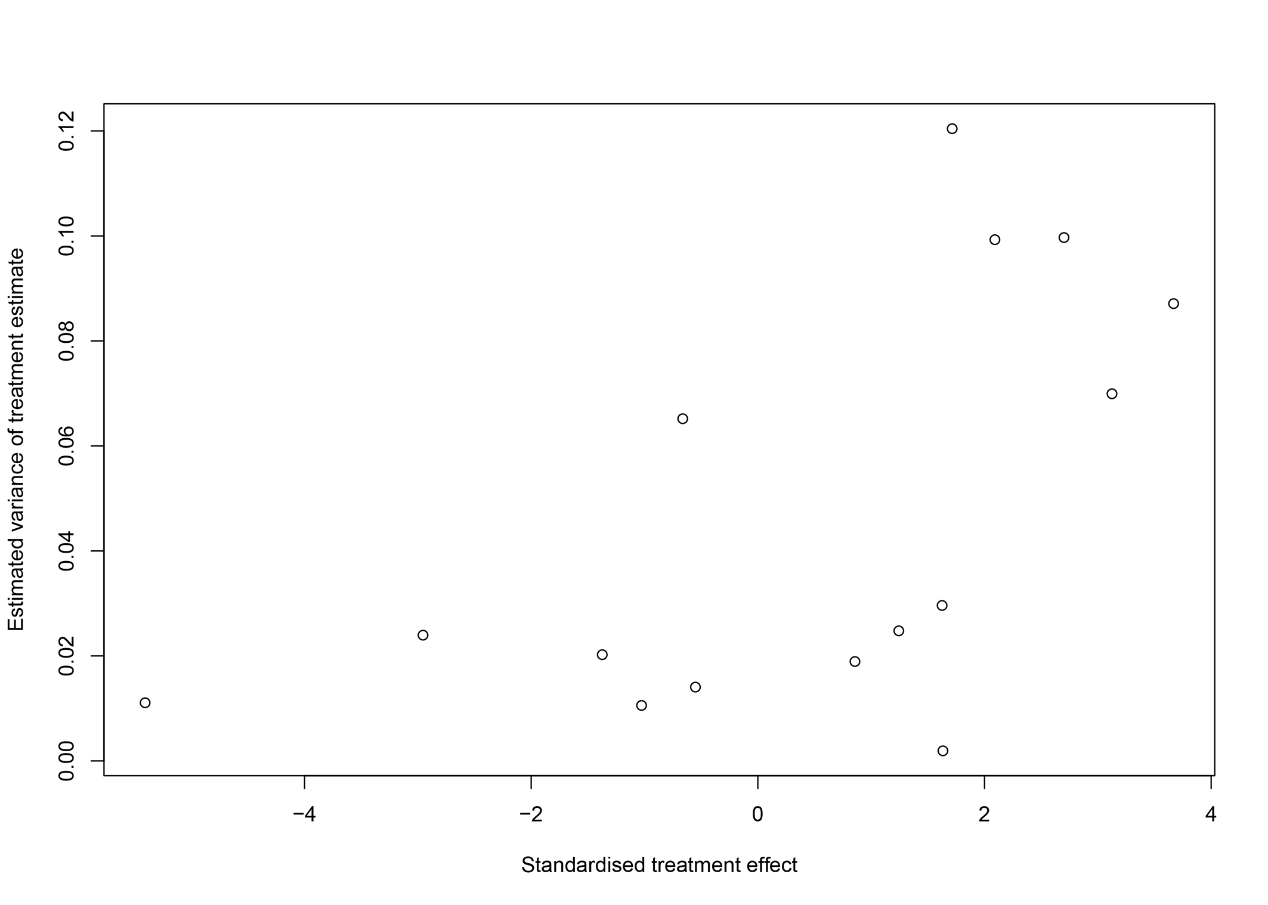


**Figure S2a, S2b.** Begg's funnel plot of urinary nickel, blood nickel and diabetes risk


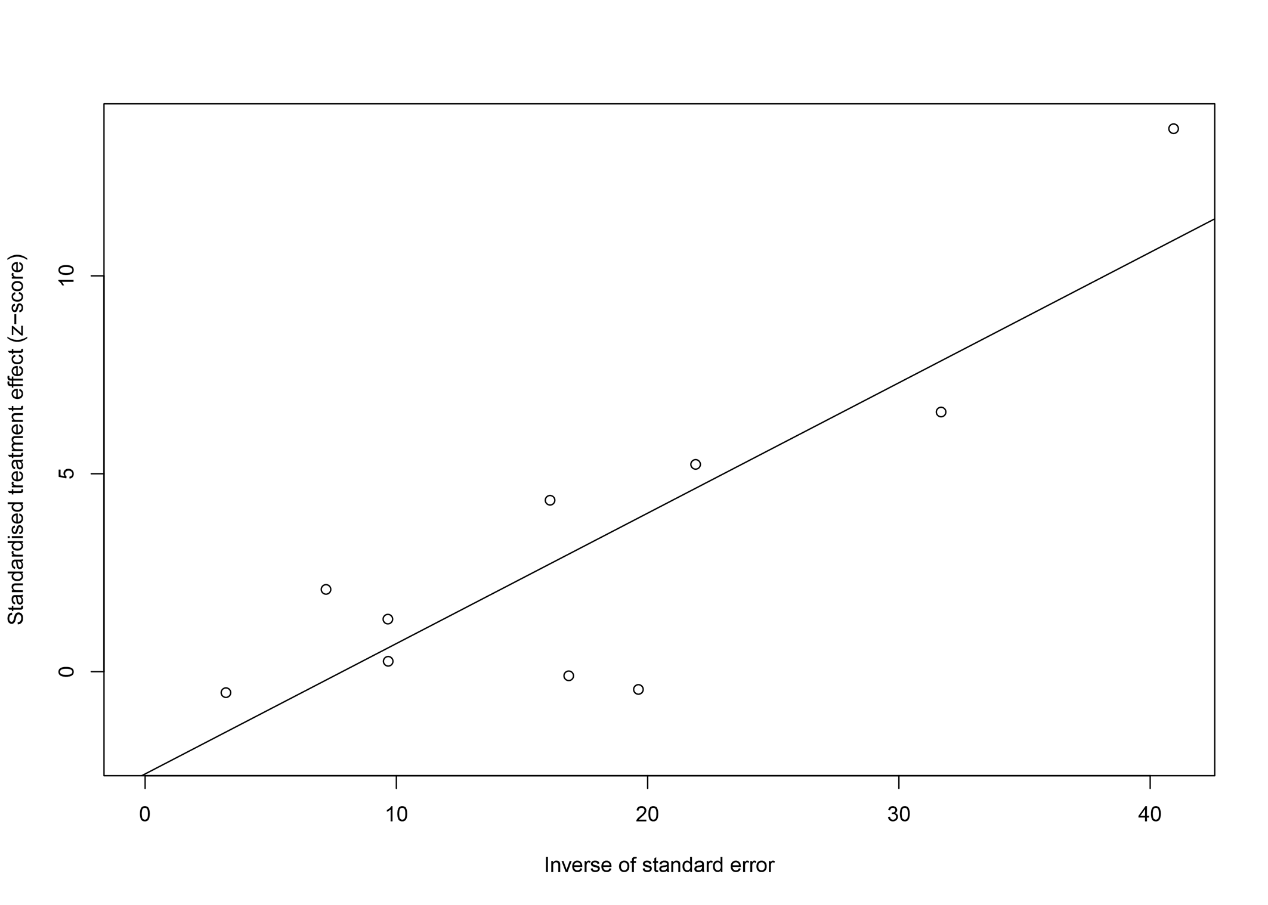


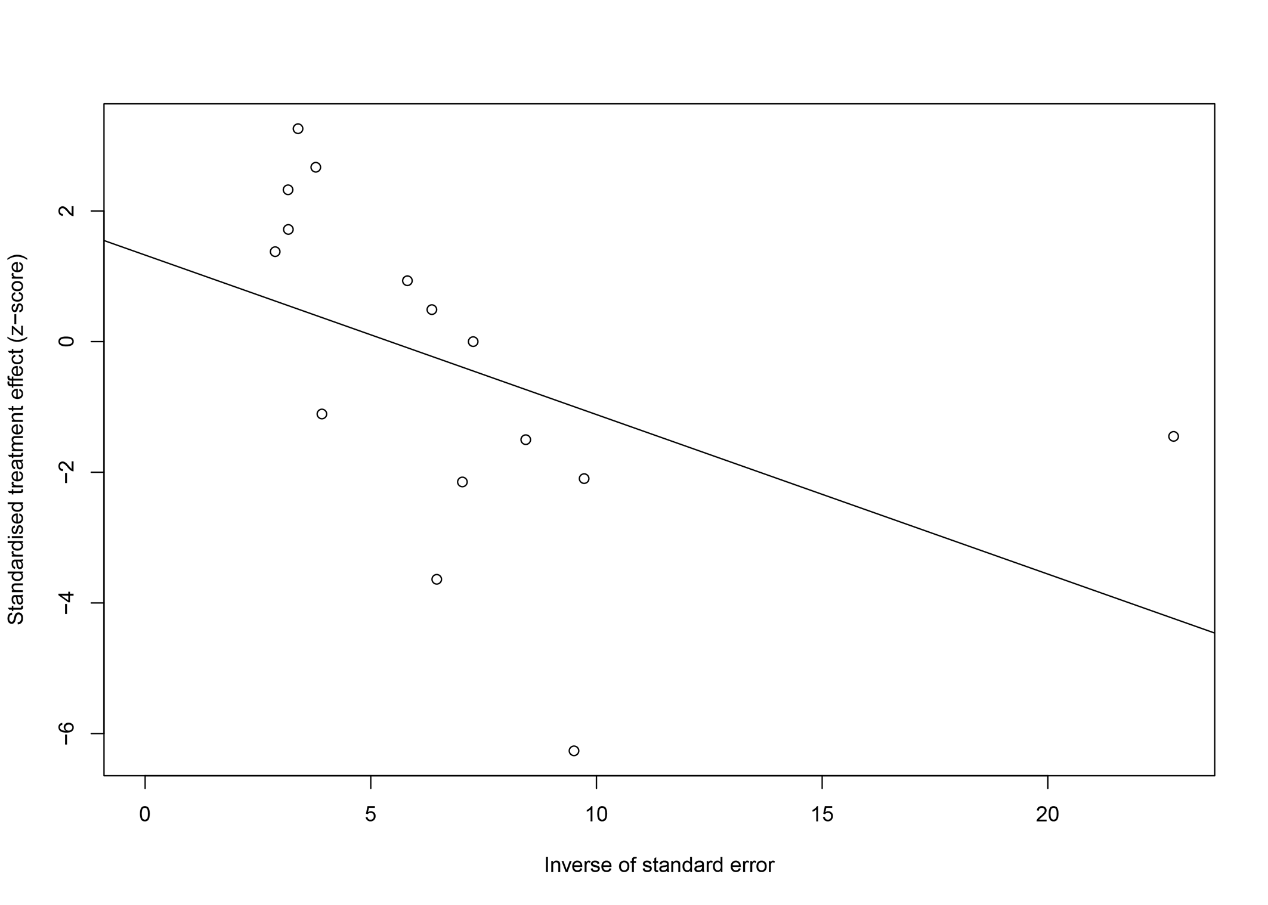

Supplement: Supplementary file 1 [file Data_Sheet_1.docx]
